# 2D wrinkle assisted zigzag plasmonic chains for isotropic SERS enhancement

**DOI:** 10.1038/s41598-025-87504-8

**Published:** 2025-01-29

**Authors:** Ziwen Yu, Swagato Sarkar, Sezer Seçkin, Ningwei Sun, Anik Kumar Ghosh, Sven Wießner, Ziwei Zhou, Andreas Fery

**Affiliations:** 1 Institute of Physical Chemistry and Polymer Physics, Leibniz-Institut für Polymerforschung Dresden e.V. (IPF), 01069 Dresden, Germany; 2 Institute of Materials Science, Technische Universität Dresden, 01062 Dresden, Germany; 3 Institute of Macromolecular Chemistry, Leibniz-Institut für Polymerforschung Dresden e.V. (IPF), 01069 Dresden, Germany; 4 Institute of Polymer Materials, Leibniz-Institut für Polymerforschung Dresden e.V. (IPF), 01069 Dresden, Germany; 5 Chair for Physical Chemistry of Polymeric Materials, Technische Universität Dresden, 01062 Dresden, Germany

**Keywords:** Template-assisted colloidal self-assembly, Plasmonic nanoparticles, Zigzag wrinkles, Plasmonic hotspots, SERS enhancement, Chemistry, Nanoscience and technology, Optics and photonics

## Abstract

Template-assisted colloidal self-assembly has gained significant attention due to its flexibility and versatility. By precisely controlling the shape of the template, it is possible to achieve custom-designed nanoparticle assemblies. However, a major challenge remains in fabricating these templates over large areas at a low cost. Recently, one-dimensional (1D) nano-wrinkle structures have been effectively used for the linear assembly of single-chain or multi-chain nanoparticles, which feature abundant interparticle nanogaps that facilitate efficient plasmonic coupling. To further enhance these assemblies by incorporating diffraction modes, we develop two-dimensional (2D) zigzag wrinkle structures that successfully assemble nanoparticles into plasmonic zigzag chains. Micro spectral measurements and FDTD simulations reveal that zigzag assemblies of plasmonic nanoparticle chains offer isotropic behavior and exhibit stronger plasmonic coupling compared to 1D assemblies, which could be highly beneficial for sensing applications. Due to the responsive PANI shell encapsulating the gold nanoparticles, this 2D zigzag assembly enables flexible tuning of plasmonic resonance under pH regulation.

**Supplementary Information:**

The online version contains supplementary material available at 10.1038/s41598-025-87504-8.

## Introduction

For the past few decades, researchers have been striving to uncover the correlations between natural structures and their properties^1–3^. Repetitive structures on micro- and nanometer scales are prevalent in natural materials and biological tissues, bestowing them with diverse functionalities such as surface adhesion, antimicrobial resistance, and optical modulation^4–7^. Notable examples, including butterfly wings, shark skin, and lotus leaves, have been extensively studied^8^. A common characteristic of these materials is their layered internal structure with nano- or micrometer-scale features, which critically determine their properties and functions^9^. To fabricate such repetitive or periodic nanostructures, researchers have identified two primary methods in the field of nanotechnology. The first one is the top-down approach, which typically employs techniques like electron beam lithography^10,11^, focused ion beam^12^, laser lithography^13^, or multi-photon lithography^14,15^ to create nanoscale features within defined, repetitive grating structures^9^. The second one is the bottom-up method, which involves the fabrication of nanostructured metasurfaces through the self-assembly of nanoscopic building blocks^16^. Such bottom-up techniques have shown great potential for scalability, efficiency, and cost-effectiveness compared to top-down methods^17^. Apart from dielectric nanospheres like polystyrene beads^18,19^, incorporating plasmonic nanoparticles into periodic arrangements^20^ opens up a new avenue of plasmonic metasurfaces with unprecedented tunability and functionality^21^. In recent years, a lot of attention has been devoted to colloidal gold and silver nanoparticles due to their unique optical and electrical properties^22–24^. As the size of metallic structures decreases, surface effects become more significant, and quantum physical effects give rise to striking optical phenomena^22^. This phenomenon, known as localized surface plasmon resonance (LSPR), arises from the collective oscillations of conduction electrons on the surfaces of the metallic nanostructures induced by the incident electromagnetic waves^25^. Gold and silver nanoparticles exhibit distinct colors in the visible and near-infrared regions due to strong LSPR quenching^26^. Therefore, arranging plasmonic nanoparticles into periodic patterns is a leading area of study in nanophotonics, significantly improving their efficiency in absorbing, directing, and distributing incoming light energy through incorporation of diffraction and photonic modes^27,28^. Side by side, developing and improving synthesis techniques remains essential, as this advancement enables the tuning of the physical properties of nanoparticles to be assembled and the control of their interactions during such assembly process^1^.

For such periodic configurations in their most simple form, plasmonic 1D lines already play a crucial role in numerous applications, including sensing^29^, structural color filtering^30^, bandgap tuning^31^, and opto-electronic devices^32^. While exploring scalable manufacturing processes at a reasonable cost, the process of forming 1D patterns through induced wrinkling on polydimethylsiloxane (PDMS) substrates emerges as a significant non-lithographic route^33–35^. This method achieves uniform pattern distribution across areas up to tens of square centimeters, effectively scaling the nanostructures to macroscopic dimensions^32,36,37^. Subsequently, these wrinkled PDMS substrates can be utilized for template-assisted self-assembly (TASA) of plasmonic nanoparticles in ordered arrays^38,39^. Of particular interest are linear chains of nanoparticles, often referred to as plasmonic polymers^40,41^. Linear assemblies of nanoparticles on 1D wrinkle structures promote efficient plasmonic coupling facilitated by abundant interparticle hotspots^42,43^. This effect, known as hotspot excitation (i.e., coherent field enhancement), can occur even between adjacent particles, depending on the interparticle distances^40,44,45^.

Although creating 1D periodic wrinkles is relatively simple, researchers have explored various methods to enhance the complexity and hierarchy of these 1D patterns and acquire more control over light-matter interactions^2,46^. To compete with the efficacy of lithographic techniques, particularly known for their capacity to generate 2D periodic structures, it is crucial to investigate the application of wrinkling methodologies toward generating 2D periodic patterns^47^. With this objective of advancing the potential of the wrinkling approach, we investigate the fabrication of zigzag wrinkled structures, commonly referred to as herringbone structures^47,48^ in the scientific community. Previously, similar two-dimensional wrinkles have also been explored by several research groups; however, their dimensions were relatively large, reaching the micrometer scale, which is clearly unsuitable for nanoparticle assembly^7,49^. Additionally, controlled wrinkle-mediated 2D patterns on polymers have also been fabricated, including several process steps and excess complexities^50,51^.

**Fig. 1:**
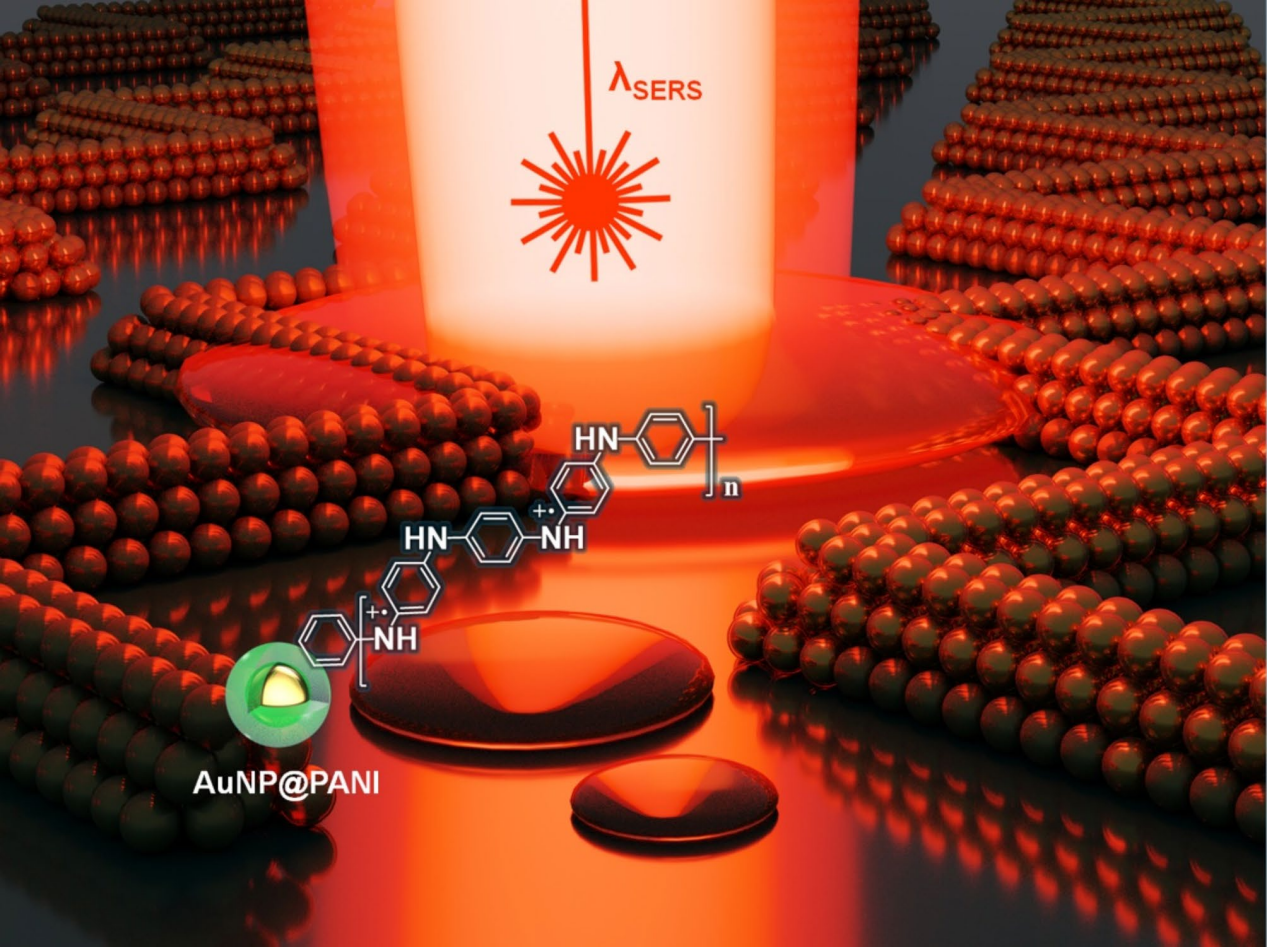
Schematic of the plasmonic system for isotropic SERS enhancement. PANI-coated gold nanoparticles (AuNP@PANI) are arranged as zigzag plasmonic chains on silica surface for enhancing SERS signals under 633 nm laser excitation.

In this study, we have employed optimized biaxial stretching and plasma treatment techniques to successfully produce nanoscale zigzag wrinkles, which facilitate the efficient assembly of plasmonic gold nanoparticles (AuNPs) into distinct zigzag chains, either within wrinkle troughs or transferred onto alternate substrates using our pattern transfer techniques. Our method overcomes major challenges in the field by integrating the advantages of plasmonic hotspots with large-scale periodic arrangements while also imparting polarization independence to exhibit an isotropic plasmonic chain response. Previous research^52^ has predominantly focused on 1D linear chains of AuNPs that provide polarization-dependent enhancements in surface-enhanced Raman spectroscopy (SERS). While alternative methods, such as assembling AuNPs into a hexagonal monolayer at the air/water interface, do allow for large-scale periodic arrangements, the attained periodicities are inherently limited by the nanoparticle shell thickness^53^. Specifically, by utilizing the optical characteristics of 2D zigzag configurations, we can achieve periodicities comparable with the plasmonic excitation wavelengths, thus enhancing lattice effects and, consequently, increasing electric field intensities. Thus, our zigzag plasmonic chains offer significant advantages in terms of scalability and enhanced isotropic optical responses, making them highly suitable for SERS measurements as sketched in Fig. 1. Additionally, the gold nanoparticles encapsulated by a responsive PANI shell grant the 2D zigzag assembly the ability to dynamically tune its plasmonic resonance under pH regulation, further broadening its potential for advanced plasmonic applications.

## Methods

### Materials

Aniline (99.5%), ammonium persulfate (98%), sodium citrate tribasic dihydrate (ACS reagent, ≥ 99.0%), HAuCl4·3H2O (≥ 99.9% trace metals basis), and sodium dodecyl sulfate (SDS, 99%) were purchased from Sigma-Aldrich. Sylgard 184 PDMS elastomer kits were purchased from Dow Corning. All these chemicals were used as received without any purification. 10% Hydrochloric acid was diluted from a 37% stock solution. The 37% Hydrochloric acid was purchased from VWR International.

### Preparation of PANI-coated AuNPs

For the AuNPs coated with a polyaniline (PANI) shell, PANI was synthesized using the chemical oxidative polymerization. 5 mL of citrate-stabilized AuNP solution (0.3 mg/mL) was centrifuged to remove excess citrate, as it could interfere with the chemical oxidative polymerization process. A concentrated citrate-stabilized AuNP solution (3 mg/mL, 0.5 mL) was mixed with aniline (2 µL), sodium dodecyl sulfate (SDS, 2.5 mL, 40 mM), and 4.5 mL of water. The mixture was vigorously shaken for 30 s, followed by the addition of 2.5 mL of ammonium persulfate (2 mM in 10 mM HCl) to initiate polymerization. After 12 h of polymerization, the PANI-coated AuNPs were purified through repeated cycles of centrifugation and redispersion in 4 mM SDS to remove unbound PANI. All chemicals were used as received. With respect to the PANI thickness controlled by polymerization time, the thickness is 6–8 nm after 12 h, 8–10 nm after 24 h, and 11–12 nm after 48 h. The thickness can be further increased to over 20 nm by repeating the polymerization process. However, in this study, the thinnest PANI shell (6–8 nm) was chosen to achieve strong plasmonic hotspots.

### Preparation of PDMS wrinkles

*1D Wrinkles*: Initially, Sylgard 184 silicone elastomer (Dow Corning) was combined with a curing agent at a 10:1 weight ratio in a plastic cup. This mixture was subjected to a two-step homogenization process in a conditioning mixer involving mixing and defoaming, each lasting 90 s at 2000 rpm. Subsequently, the homogeneous prepolymer was uniformly poured into a square plastic box measuring 10 × 10 cm² and left on a level surface for two to three days to ensure complete air bubble removal. The mold was then transferred to an oven and cured at 80 °C for four hours, followed by cooling to room temperature on a flat surface. The solidified PDMS was cut into rectangular strips, each measuring 1 cm in width and 4.5 cm in length, using a surgical blade. These strips were mounted on a custom-built unidirectional stretching device to apply a 40% stretch. Post-stretching, the strips were exposed to oxygen plasma for a defined duration at 100 W and 0.3 mbar of oxygen pressure, followed by cooling to ambient temperature. The final step involved the gradual mechanical release of the stretching force, forming the wrinkled PDMS templates, which were finally cut into 1 × 1 cm² pieces for experimental use.

*2D zigzag wrinkles*: The preparation of 2D zigzag wrinkles followed the same steps as for the 1D wrinkles, except that the prepolymer was poured into a different mold instead of the plastic box. The cross-shaped PDMS was gently removed from the mold using tweezers, followed by mounting on a more specialized custom-built bi-directional stretching device. To create the 2D wrinkles, equal or different stretch ratios (15% or 20%) in both directions were applied. After plasma treatment, the PDMS was cooled to room temperature. The final step was to evenly and slowly release the stretching force (the releasing speed was approximately 2–4 mm/min). The stretching in both directions was released sequentially to avoid uneven stress on the PDMS. Rapid release of the stretching force could result in unwanted cracks.

### Wrinkle-assisted assembly of nanoparticles

*Spin-coating on PDMS Wrinkles*: Initially, the synthesized AuNP@PANI solution was subjected to sonication at 20 °C for 30 min to prevent nanoparticle aggregation. For the arrangement of nanoparticles into desired structures, both 1D linear and 2D zigzag PDMS wrinkles were employed as templates. Prior to spin-coating, these templates were hydrophilized by immersion in 10% hydrochloric acid for one hour. 4 µl droplets of nanoparticle suspension (C_Au0_=18 mg/mL) were manually spread to the PDMS wrinkle templates. The spin-coating was executed in two stages: initially at 222 revolutions per minute (rpm) for two seconds, followed by a longer duration of 90 s at 1600 rpm. Throughout this process, the suspension progressively thinned and extended toward the edges of the templates.

*Confinement self-assembly using PDMS wrinkles*: This technique facilitated the assembly of plasmonic nanoparticles into desired geometries on various substrates following similar patterns present on the templates used. Specifically, a silicon wafer was selected as the substrate for this study. The surface of the silicon wafer was rendered hydrophilic via a plasma treatment that was conducted for 90 s at 100 watts and an oxygen pressure of 0.3 millibars. Subsequently, 4 µl droplets of the same PANI-coated Au nanoparticle suspension (C_Au0_=18 mg/mL) used in the spin-coating assembly were drop-cast onto the silicon wafer. Both 1D linear and 2D zigzag wrinkles were positioned over the wafer, permitting the nanoparticles to self-assemble, influenced by gravitational forces exerted by the wrinkles.

### Surface characterization of wrinkles and assemblies

The morphology of the as-prepared 1D and 2D zigzag wrinkles and nanoparticle assemblies was characterized using atomic force microscopy (AFM) and scanning electron microscopy (SEM). AFM imaging was performed in the air with a FastScan Bruker system in ScanAsyst mode. SEM imaging was performed with a NEON 40EsB microscope in secondary electron (SE2) mode.

### Finite-difference time-domain simulation

For the Finite-Difference Time-Domain (FDTD) analysis, a commercial-grade electromagnetic solver by Ansys Lumerical^54^ was employed, operating under normal incidence with a broadband source (Bloch/Periodic) spanning from 400 to 1000 nm. The injection axis of the incident light was kept in the -Z direction with periodic boundary conditions along the X and Y axes and perfectly matching layers along the Z axes. The simulations were conducted using an automatic non-uniform mesh with the smallest mesh step set to 0.25 nm and an FDTD background index of 1. The optical characteristics of these AuNPs were derived from data provided by Johnson and Christy^55^ with a dielectric coating of 1.5 representing the thick PANI shells. Transmittance (T) and extinction characteristics (calculated as Ext = − ln T) of various configurations such as Zigzag chains, linear chains, and randomly distributed AuNPs were determined using frequency-domain field and power monitors. The polarization of the electric field varied from − 90° to 90°, covering a total span of 181 degrees. Additionally, electric field distributions were recorded with frequency-domain field profile monitors using a single-wavelength source of 633 nm and a 2-nm mesh overlay throughout the cross-sectional particle plane touching the substrate. The substrate index was considered as 1.45, representing the glass surface. For angle-resolved calculations, the broadband source was changed from ‘Bloch/Periodic’ to ‘BFAST (Broadband Fixed Angle Source Technique)’.

### UV-Vis-NIR spectroscopy

An ultraviolet-visible–near-infrared (UV-vis-NIR) spectrophotometer (Cary 5000, Agilent Technologies), set in a transmission configuration, is employed to measure optical responses across a spectrum ranging from 400 to 1000 nm. A rotatable polarizer is utilized to explore the impacts of both s-pol (transverse electric, TE) and p-pol (transverse magnetic, TM) polarizations. In general, the electric fields for s-pol and p-pol are perpendicular and parallel to the plane of incidence (POI) while considering oblique incidences, respectively. However, for normal incidences, as carried out in this research, ‘s’ and ‘p’ represent 0° and 90°orientation of the electric fields concerning the linear 1D chains.

### Surface-enhanced Raman spectroscopy

SERS and reference Raman spectroscopy measurements were conducted using the Renishaw inVia Qontor Confocal Raman Spectrometer with a backscattering configuration (Gloucestershire, UK). The experiments utilized a Leica microscope equipped with a 20x magnification lens and NA of 0.45 (Leica Microsystems GmbH, Wetzlar, Germany). The 633 nm (532 nm) and 785 nm excitation wavelengths were used with corresponding laser powers of 290 µW (860 µW) and 5,9 mW, respectively, and acquisition times of 1 s and 5 s. The Raman signals collected were dispersed using gratings of 1800 l/mm and 1200 l/mm, then captured by a CCD detector. To explore the isotropic effects, Raman measurements were taken from the same spot by rotating the AuNP patterned substrates at discrete angles of regular intervals. These exact locations were recorded using a motorized stage for consistent repositioning in subsequent measurements. For SERS mapping, an x20 (×100) objective with an NA of 0.45 (0.75) was employed to scan the defined area. For this purpose, the integration time was adjusted to 0.5 s (0.1 s). Additionally, the distance between each step was set to 0.5 μm (0.1 μm). By fitting the Raman peaks of the PANI, the false color mapping of the scanned area was prepared according to the intensities of the fitted Raman mode at 1336 cm^− 1^ wavenumber. Raman spectrum baseline corrections were carried out using WiRE 5.6 software.

## Results and discussions

### 1D wrinkle-assisted assembly of gold nanoparticles

In this section, we explore the results concerning the 1D linear assembly of plasmonic nanoparticles, following the protocols outlined in the methods section. The assembly of core-shell particles requires PDMS wrinkles of the corresponding size for successful incorporation into the troughs of the wrinkled PDMS surface. Both the concentration of the particles and the rotational speed significantly influence the spin-coating mediated assembly process. Recent advancements in traditional 1D wrinkle templates have paved the way for the linear assembly of various plasmonic nanoparticles, as discussed in the introduction section. The minimal gaps between adjacent particles amplify plasmonic excitation through the formation of plasmonic hot spots that can be utilized in polarization-sensitive SERS signal enhancement. Building upon this foundational concept, we move a step further to incorporate sharp features into the wrinkled assembly of AuNPs to enhance plasmonic hotspots as well as isotropic signal enhancement. For this, we discuss the successful fabrication of 2D zigzag wrinkles along with controlling their dimensions to match nanoparticle sizes for successful integration and pattern transfer.

### Effect of parametric alteration on 2D wrinkle formation

**Fig. 2:**
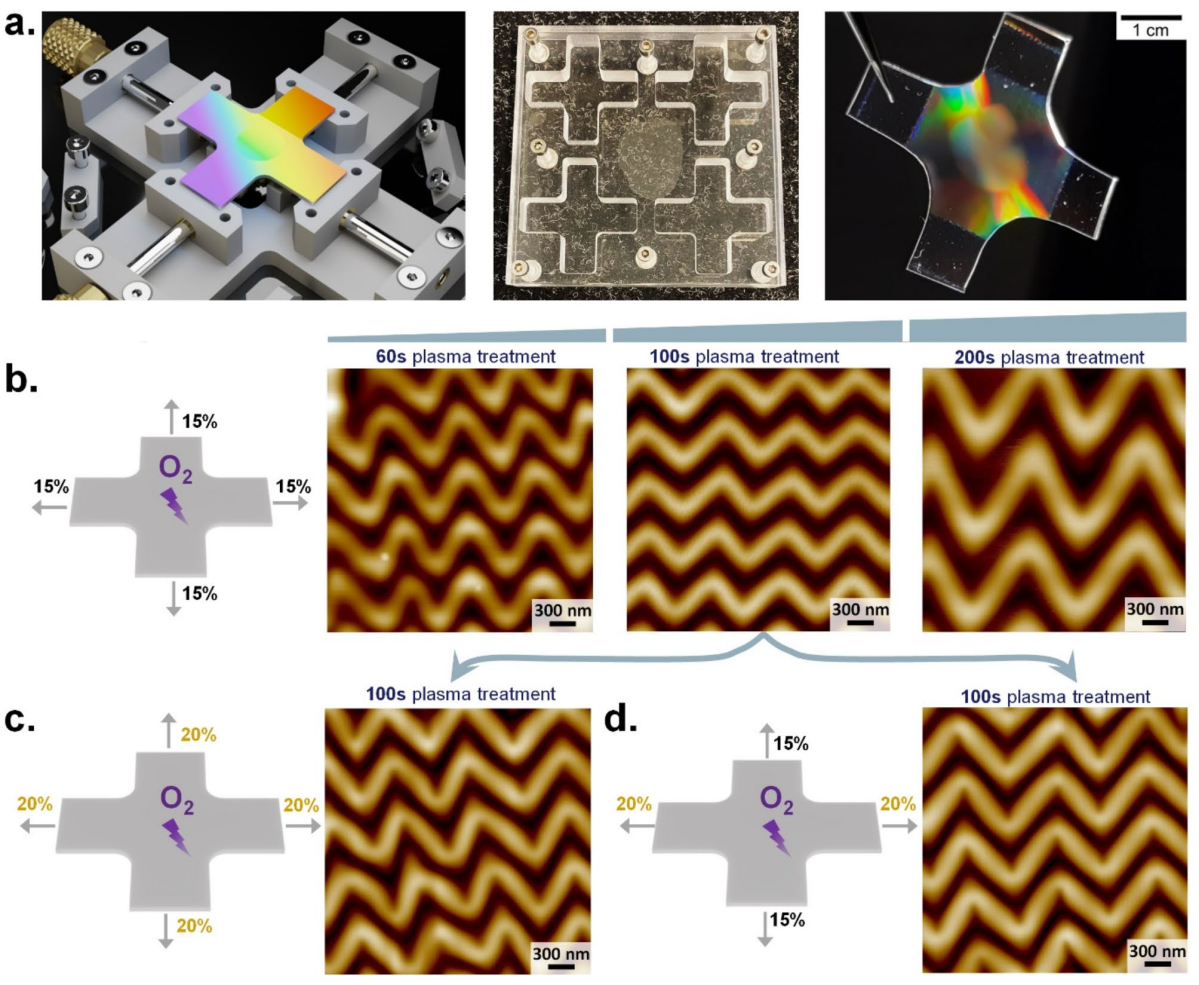
Effect of parametric alterations on 2D wrinkling. **a** Schematic of 2D wrinkled substrate fabrication by loading ‘cross-shaped’ PDMS substrate into a bi-directional stretching device. The photograph of the relevant mold and the final 2D wrinkled PDMS substrate after strain release are also shown. **b.** Effect of plasma treatment time in case of symmetric stretching (15%) with AFM profiles for 60 s, 100 s, and 200 s. **c**. Sketch of increased strain (symmetric, 20%) for 100 s plasma treatment with the resulting AFM profile. **d.** Asymmetric strain (15% and 20%) for 100 s plasma treatment, resulting in the desired zigzag patterning as confirmed from the AFM image. The 3D models were created using Blender version 4.0. Link: https://www.blender.org/download/.

Unlike the unidirectional stretching used to form 1D wrinkles, 2D wrinkles involve stretching in both parallel and perpendicular directions, either symmetrically or asymmetrically, resulting in a zigzag wrinkle structure. A bi-directional stretching device was required to produce strain along two different axes of a ‘cross-shaped’ PDMS substrate to generate the 2D wrinkled pattern. Figure 2a shows the schematic of the stretching device that enables the possibility of producing bi-directional strain along the two orthogonal arms of the ‘cross-shaped’ PDMS substrate. Side by side, actual photographs of the such specific designed mold (where four substrates can be prepared at once for every PDMS curing) and the finally produced PDMS wrinkles following the plasma treatment and strain release are also provided. As seen from this image, the central section can be identified as a 2D patterned region with edges still having 1D patterning that shows comparatively brighter diffraction patterns. To confirm this 2D nature at an initial stage, we have carried out bright-field microscopic imaging and diffraction order collection following a Bertrand lens setup^35^, images of which are shown in Figure S1. Furthermore, AFM images were obtained to investigate the surface characteristics in detail. We have concluded that three important parameters can significantly affect the 2D output, namely the plasma treatment time, the stretching ratio, and the strain release sequence. To have a better understanding of how these parameters affect the formation of 2D wrinkles, we have adjusted one parameter at a time while keeping others constant.

*Plasma treatment time*: First, we found that increasing plasma treatment time leads to larger and more homogeneous 2D wrinkles. Producing smaller-sized zigzag wrinkles remains challenging but is essential for nanoparticle assembly. Figure 2b shows the uniform bi-directional stretching with 15% strain executed on both axes of the PDMS substrate with corresponding AFM results for varied plasma treatment times of 60s, 100s, and 200s.

*Stretching ratio*: Because of the two stretching directions involved in fabricating 2D wrinkles, one can have further flexibility in controlling the wrinkle structure by executing both symmetric (Fig. 2c) and asymmetric stretching (Fig. 2d) along the two axes. Considering the symmetric stretching, we observe that increasing the strain from 15 to 20% results in larger and higher wrinkles, as demonstrated in AFM images. For the asymmetric stretching ratios, we gradually vary the force in a single direction to observe the effects of different stretch ratios on the wrinkles. By comparing the symmetric and asymmetric ratio, we observe that under the same plasma treatment time of 100s, asymmetric strain contributed to narrowed wrinkle troughs, which is beneficial for nanoparticle assembly. All the AFM images are considerd with dimensions of 3 micrometers for this systematic comparison.

*Sequence of strain releasing*: Finally, the strain release sequence for asymmetric stretching plays a crucial role in wrinkle orientation for the 2D wrinkles. Releasing the smaller strain first results in inhomogeneous wrinkles, likely due to the shear stress from larger stretching disrupting the smaller stretched wrinkles. Additionally, there is a limit to the stretch ratio; when the difference between stretch ratios is too large (e.g., 30% and 15%), significant cracks can form, indicating the PDMS surface has begun to tear. These results are shown in Figure S2, with AFM images showing scans over 10 micrometers. Based on our experiments, 2D wrinkles with moderate plasma treatment time of 100 s and 20%-15% stretch ratios were selected as templates for gold nanoparticle assembly, as discussed in the upcoming section.

### 2D zigzag wrinkle-assisted assembly of gold nanoparticles

**Fig. 3:**
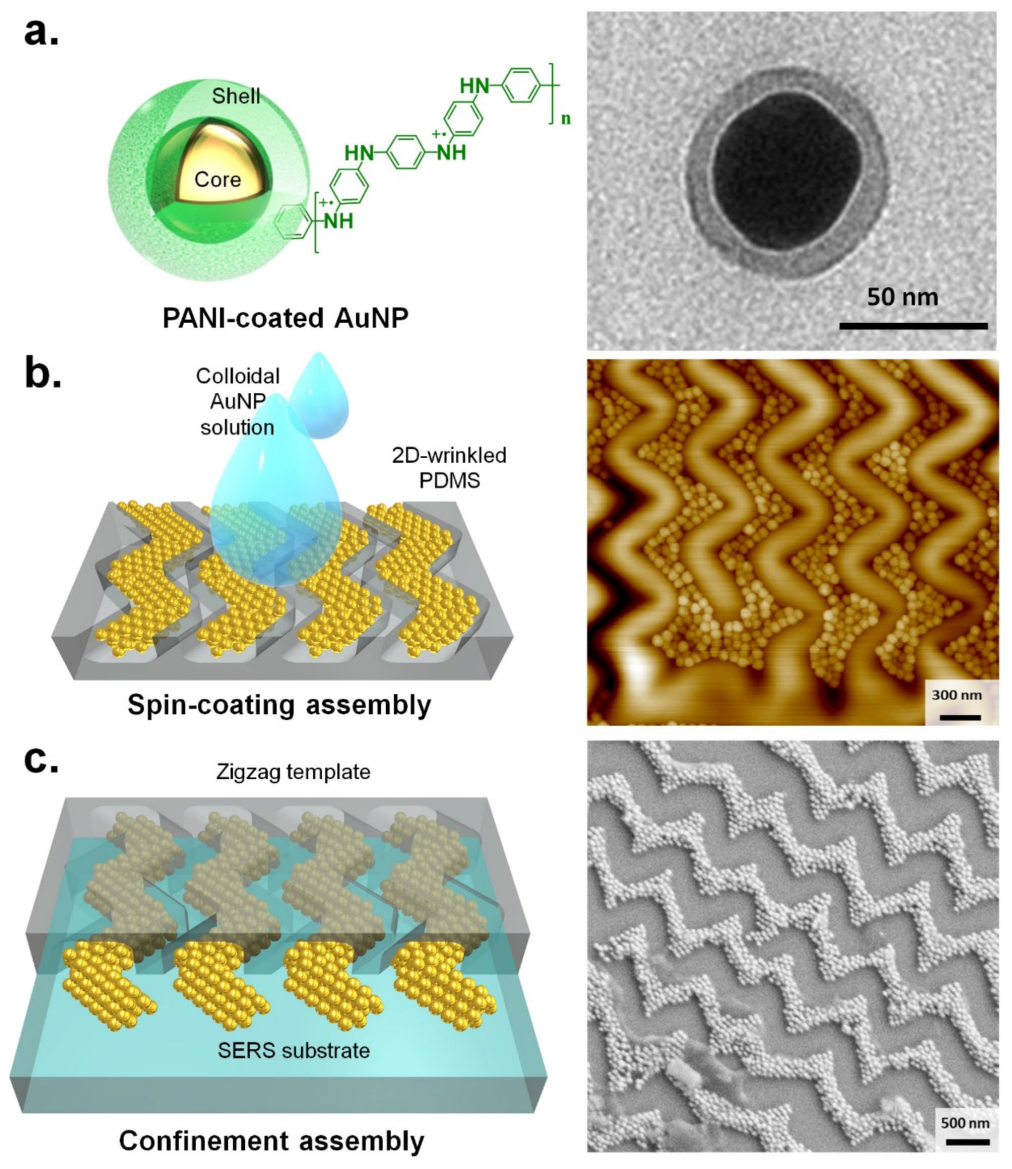
Assembly of AuNPs in zigzag chains. (**a**) Schematic of PANI-coated AuNPs with a core diameter of 50 nm and shell thickness of 6–8 nm. The TEM image in the right pabel shows an isolated PANI-coated AuNP. (**b**) Assembly of AuNPs within the PDMS wrinkling through spin coating. AFM images reveal a closed-packed assembly of AuNPs within the zigzag troughs. (**c**) Confinement-driven self-assembly using the wrinkled PDMS substrate as a mold, enabling the transfer of AuNPs onto a target substrate to form zigzag chains. The 3D models were created using Autodesk 123D software (Autodesk Inc., 2015).

Compared to top-down techniques like e-beam lithography and ion-beam milling, creating a plasmonic array with the assistance of 2D wrinkles is convenient in terms of large areas coverage (on the centimeter scale or larger) and their feasibility for practical applications. For the plasmonic blocks, we have chosen polymer-coated colloidally stable gold nanoparticles. Figure 3a shows the schematic of the PANI-coated AuNPs with the corresponding TEM image. These PANI-coated AuNPs, with an overall diameter of around 70 nm (the thickness of the PANI shell is approximately 6–8 nm, Figure S3), are utilized for assembling in the targeted Zigzag fashion. For this, we have explored two methods. The first is the direct spin-coating of these AuNPs onto the 2D wrinkled PDMS substrate. Spin-coating has been a viable approach for coating nanoparticles over both non-patterned^56,57^ and patterned substrates^58^. Using PDMS wrinkling, followed by spin-coating, Hanske et al. have significantly achieved plasmonic nanoparticle chains that have been wet transferred into target substrates^59^. The second.

method is a more direct approach to assembling the nanoparticles into the target substrate, i.e., by employing confinement self-assembly^60^. In this method, a concentrated colloidal AuNP@PANI solution is drop-cast on a substrate, and the PDMS template is subsequently imprinted^61^. Significantly, the technique of confinement self-assembly emerges as a highly adaptable method in nanofabrication, not merely confined to the construction of plasmonic nanoparticles but also applicable to the arrangement of considerably smaller semiconductor quantum dots^62,63^. Figure 3b shows the schematic of the assembly process via spin-coating with assembled AuNPs in zigzag troughs of the PDMS substrate, as confirmed via the AFM profile. The confinement self-assembly scheme and the SEM image of the resultant Zigzag AuNP chains on a silicon substrate are shown in Fig. 3c. Compared to 1D assembly, which primarily produces single linear chains with gaps, 2D zigzag assembly creates multi-chain structures with excellent coverage. The narrow gaps between nanoparticles enhance plasmonic hotspots, making the 2D zigzag structure particularly effective for sensing applications. Additionally, the increased number of assembled nanoparticles further enhances the plasmonic coupling effect. Particularly, the confinement assembly approach has been utilized to explore AuNP patterned substrates in different configurations for the isotropic SERs enhancement applications.

### Optical characterization of zigzag chains, linear chains, and randomly distributed AUNPs

**Fig. 4:**
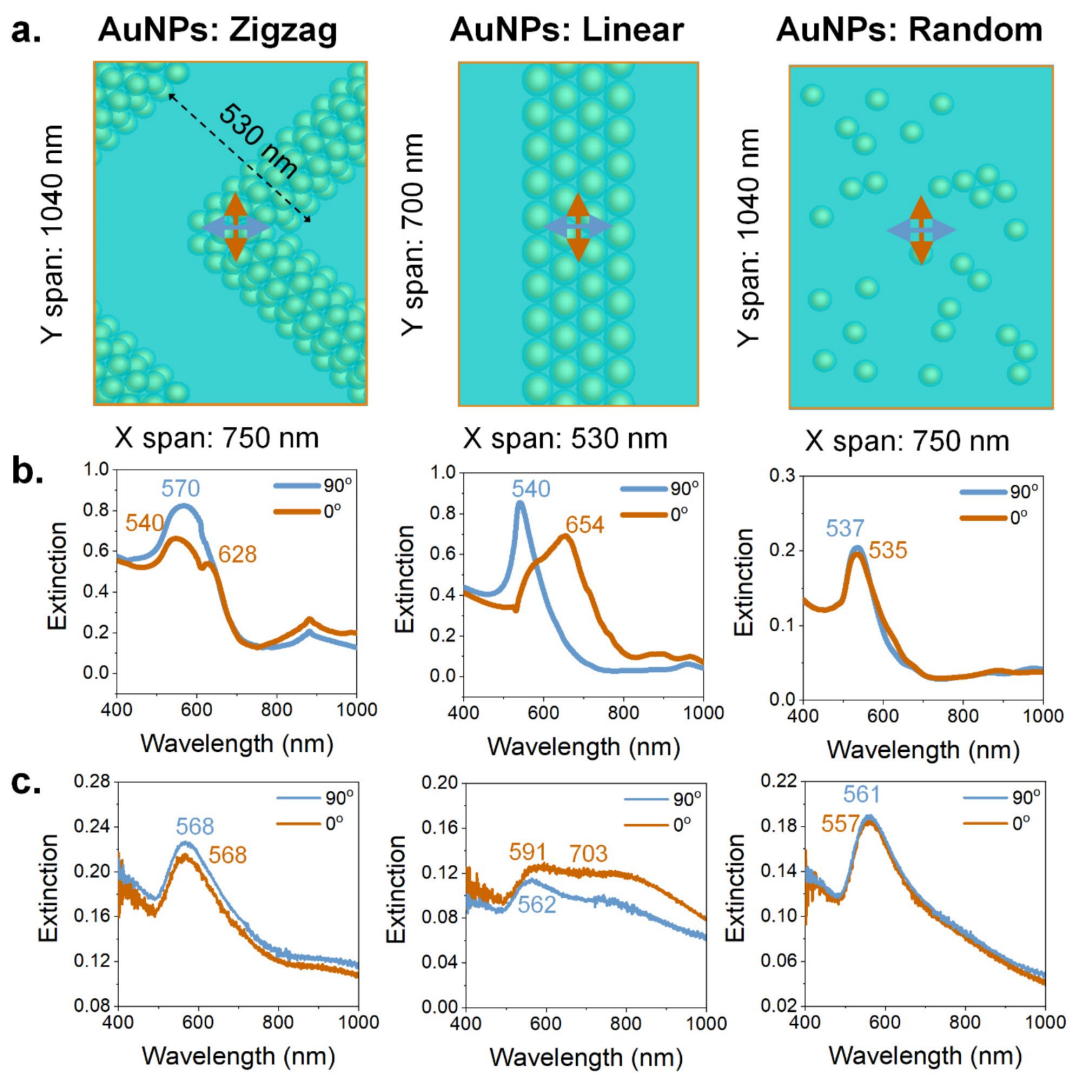
Comparative analysis of extinction spectra. (**a**) FDTD modeling approach illustrating a single repetitive unit to evaluate polarization-dependent extinction spectra for zigzag chains, linear chains, and randomly arranged AuNPs. (**b**) FDTD-calculated extinction spectra for these three configurations under two orthogonal polarization states. (**c**) Experimentally obtained extinction spectra corresponding to the three configurations.

In order to explore the plasmonic modes associated with the assembled chains, we have both numerically and experimentally characterized the zigzag configuration, along with a comparison with other cases, as shown in Fig. 4. Here, the targeted 2D zigzag plasmonic chains are compared with a 1D linear configuration, and randomly distributed isolated nanoparticles are also evaluated. For modeling the zigzag AuNP chains, the parameters are directly taken in accordance with the experimental results shown via the SEM image in Fig. 3c, where the periodicity along the X-axis is around 750 nm. As seen from the SEM image, the PANI-coated AuNPs are arranged in a hexagonal closed-packed (HCP) structure where the spherical nanoparticles are also placed in the form of layers. Hence, for the modeling purpose, we have considered a 3-layered system where the base, intermediate, and top layers contain 4, 3, and 2 particle-based chains, respectively (Figure S4). Apart from the hexagonal arrangement in the base layer, arrangement along the Z-direction is also HCP, i.e., ‘A-B-A’ type positioning of the individual dielectric-coated gold nanospheres. The unit cell with an X span of 750 nm and a Y span of 1040 nm, resembling a rotated ‘L’-shape (with equal arm length), is shown in the left panel in Fig. 4a, which can be repeated in X and Y directions to recreate the array of the zigzag patterning (Figure S5). Here, we have considered the angle between the upper and lower arm (within the unit cell) to be 90°. For comparison of the zigzag patterning with conventional 1D confinement assembly^52,64^, we have also considered a single-layered linear AuNP chain, as shown in the center panel of Fig. 4a. However, to compare with the Zigzag patterning, the periodicity considered for the FDTD unit cell for such linear plasmonic chains is also kept at 530 nm. Side by side, to demonstrate the effect of uniformly distributed AuNPs in comparison to the zigzag chains, we have used randomly positioned dielectric-coated gold spheres, as shown in the right panel of Fig. 4a.

For all three cases, the AuNPs are modeled to be placed on a glass substrate. The extinction spectra for these three cases are obtained from the FDTD calculated transmittances while considering the two orthogonal polarization states, as shown via the two differently colored arrows within the schematics of the simulation span. For the zigzag AuNP chains, both 0° and 90° polarization can have equal components split to excite the upper arm and lower arm of the unit cell. This results in the excitation of almost identical extinction spectra, as shown in Fig. 4b (left panel). The 0° polarization, parallel to the X-axis, generally excites plasmonic longitudinal chain modes for linear AuNP chains oriented along the Y-axis^59^. However, the AuNP core diameter (apart from the dielectric shell) and the interparticle distance between two adjacent gold cores play a significant role in determining the position of the resonant wavelength. For such linear chains, the orthogonal polarization state 90° excites single-particle resonant-like modes. This contrast between the two polarization states can be distinctively seen in Fig. 4b (center panel). For these two periodic cases (2D zigzag and 1D linear), one can also observe diffraction-mediated Rayleigh Anomaly (RA)^65^ that appears as a peak/dip in the extinction spectra. For a generalized 2D lattice with an orthogonal basis, the positions of the RA_S/C_ are given by $\:{\lambda\:}_{RA}={n}_{S/C}{\left[\frac{{m}^{2}}{{{P}_{x}}^{2}}+\:\frac{{n}^{2}}{{{P}_{y}}^{2}}\right]}^{-\:\frac{1}{2}}$, where P_x_ and P_y_ are the periodicities along the X and Y axis, m and n are the diffraction orders, with n_S/C_ representing the substrate/ cover index. Thus, the dip and peak near 600 nm and 900 nm in Fig. 3b, i corresponds to RA_C_(± 1, ± 1) and RA_S_(± 1, ± 1), respectively. Similarly, for the 1D lattice in Fig. 3b, ii, the dip and peak near 530 nm and 750 nm correspond to the RA_C_(± 1, 0) and RA_S_(± 1, 0), respectively.

For the randomly distributed particles, both 0° and 90° polarization exert almost identical spectra. The little discrepancies (537 nm vs. 535 nm) found between these two polarization states are because of the particle cluster orientations specific to the.

polarization axes. This gives the idea that the arrangement of clusters plays a significant role, which is also one of the reasons for the discrepancies between the two polarization configurations in the zigzag case. One can readily observe from the linear 4-particle chains (Fig. 4b, center panel) that the longitudinal modes appear at around 654 nm, whereas the single-particle-based cluster mode appears at 540 nm. For the zigzag chains, the 0° shows both the cluster modes (540 nm) and chain modes (628 nm), whereas, for the 90° case, these two have merged to form a cluster effect at 570 nm. Nevertheless, the experimental counterparts shown in Fig. 4c also show similar trends. The linear chains have distinctly different extinction spectra, whereas the zigzag and random particle distributions have almost similar spectra for excitation with two orthogonal polarizations. However, from the point of view of plasmonic hotspots, the zigzag chain modes would have additional benefits compared to the randomly distributed AuNPs, which are discussed in the upcoming sections.

**Fig. 5:**
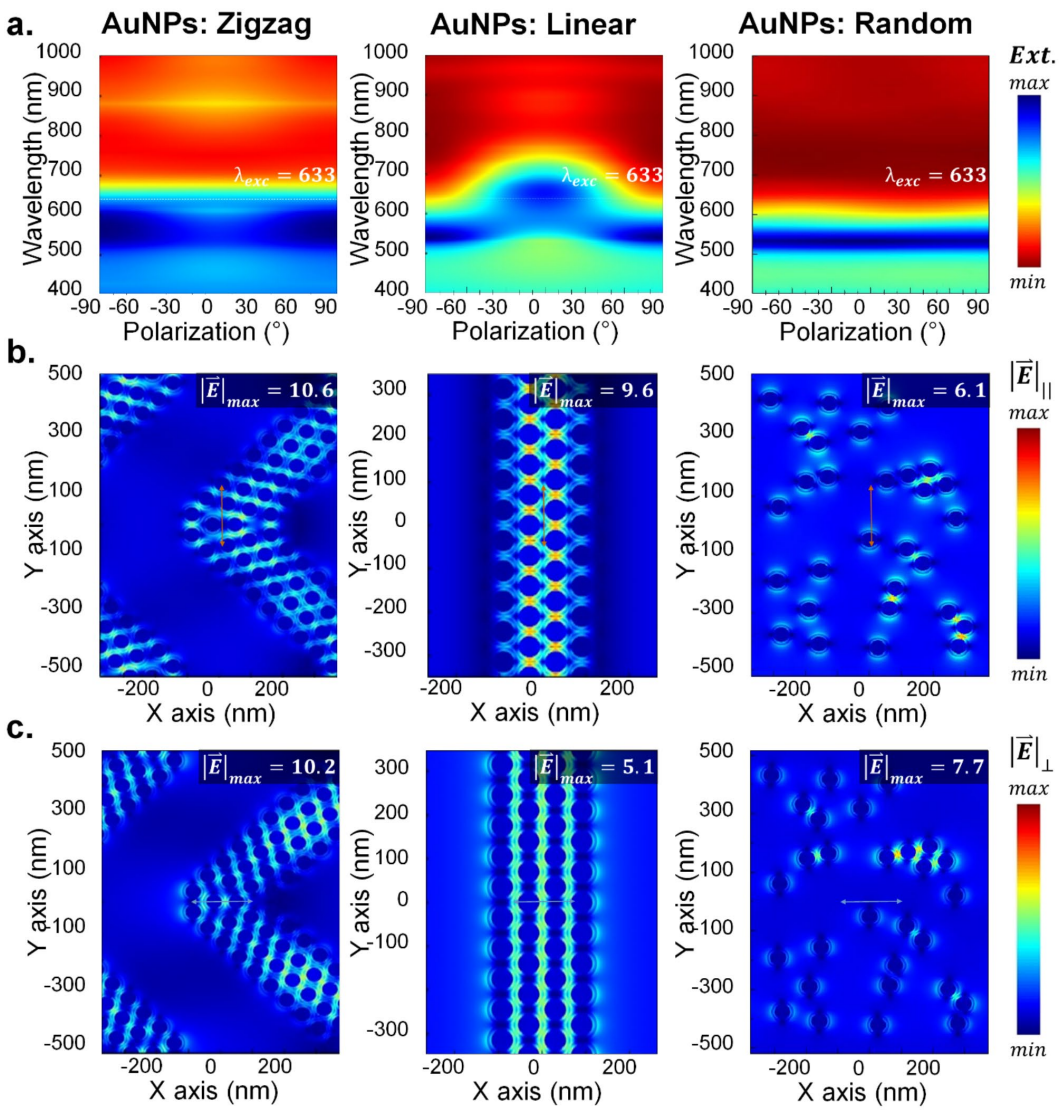
FDTD-based comparison for isotropic excitation mechanism. (**a**) Polarization scan from − 90° to 90° for zigzag chains, linear chains, and randomly arranged AuNPs in discreet steps of 1°. (**b**) Calculated electric field profile for these three configurations at 0° polarization. (**c**) Similar field profiles for 90° polarization.

The polarization dependence of linear AuNP chains and polarization independence of zigzag AuNP chains, as well as randomly distributed AuNPs, are further evaluated using FDTD simulation. Instead of the two cross polarizations 0° and 90°, a full polarization-sweep from − 90° to 90° is carried out in discreet steps of 1° to provide a high-resolution angle-dependent extinction response of these three cases. Figure 5a shows these extinction spectra as a function of these 181 degrees for the zigzag, linear, and random cases. While the zigzag and random cases have consistent spectra across the polarization span, the noticeable difference between them lies in the position of the extinction peak; the zigzag chains have a larger wavelength span corresponding to the plasmonic coupled modes of chains and oligomers^40^. One can also find that the position of the RA_S/C_ remains unaffected by such polarization sweep. The distinct difference is found for the linear chain case where − 90° and 90° represented the cluster/oligomer modes, and 0° represented the chain modes at higher wavelengths.

In order to see the effect of field confinement for excitation with a He-Ne laser, these three different configurations are excited with a 633 nm single wavelength source. The corresponding electric field distribution is recorded for both the 0° (Fig. 5b) and 90° (Fig. 5c). As expected, the zigzag case has shown an almost identical enhancement factor. In contrast, the linear case has a significant difference because the chain modes appear nearer to the laser excitation wavelength. Due to the lack of plasmonic hotspots for the random case, the field enhancement is relatively lower and does not have identical values for both polarization orientations. We have also taken the liberty of constructing a uniformly distributed hexagonal closed-packed single-layered AuNP array that spans over the entire FDTD cell, with promises of isotropic behavior, and compared with over such random cases to highlight any benefits, if any. Such hexagonally closed pack assemblies have been recently reported using poly(N- isopropylacrylamide) particle coating to move AuNP monolayer from a water-air interface directly onto a substrate, facilitating the creation of 2D plasmonic gratings^66^.

Figure S6 shows the FDTD span, extinction line spectra, polarization sweep, and electric field enhancements for such hexagonally arranged uniform monolayer in comparison to randomly distributed particles. It is seen that while such monolayers may support polarization isotropic behavior, the plasmonic hotspots and field enhancements are higher for the periodic arrangements, which are advantageous for SERS measurements for coupling the diffraction-mediated RA modes with plasmonic extinction^52^. To highlight the importance of the 2D zigzag and 1D linear lattices over randomly/uniformly distributed particle assembly, we have carried out angle-dependent extinction calculations along both the X and Y-axes for all these cases. Figure S7 shows further coupling possibilities between the RA and plasmonic modes at a higher angle of incidence (AOI) only for the 1D linear /2D zigzag assemblies.

### Comparative study for the SERS enhancements

**Fig. 6:**
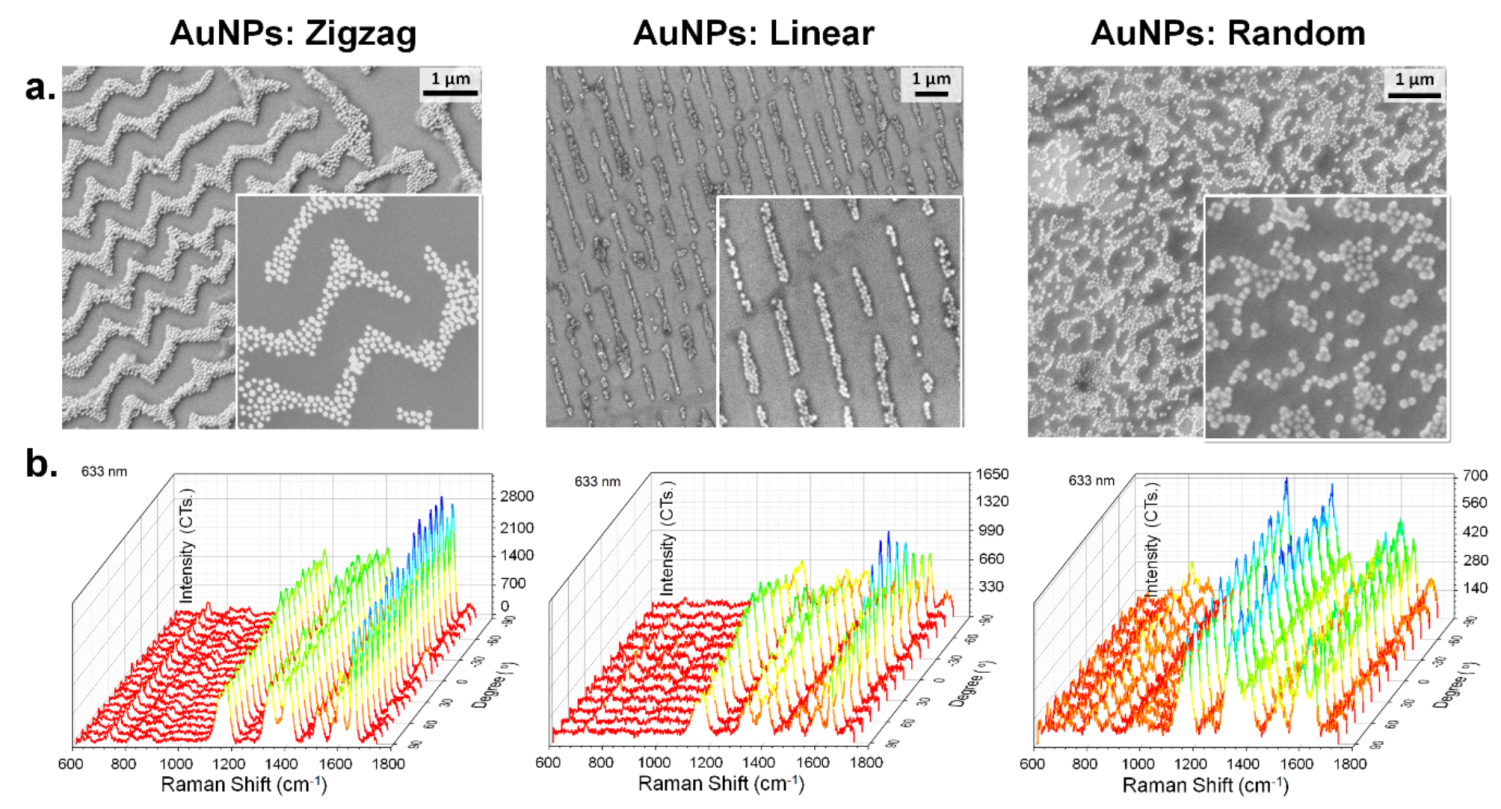
Comparative analysis for SERS enhancement. (**a**) SEM images showing the three configurations: zigzag chains, linear chains, and random arrangements of PANI-coated AuNPs under laser excitation of 633 nm for their performance as isotropic SERS substrates (**b**) Raman shifts from these three corresponding configurations, plotted as a function of sample stage rotation.

To compare the SERS performance, all three cases of 2D-zigzag, 1D-linear chain, and random particles are experimentally evaluated for signal enhancement toward the detection of the PANI coating. For these, the three configurations are excited with a laser emitting at a wavelength of 633 nm. Figure 6a shows the SEM image of these SERS substrates with plasmonic nanoparticles arranged in 2D zigzag, 1D lines, and random distributions. While the inset shows the magnified version, arrangements spanning over large areas with uniform coverage are provided in Figure S8. The impact of the polarization angle on the SERS signal is examined by rotating the sample from − 90° to 90° in increments of discreet steps (Fig. 6b). To prevent any inhomogeneous distribution of analyte materials or the orientation effect of the analyte molecules^67,68^, the Raman modes of the ligand polymer (PANI) covering the plasmonic nanoparticles are investigated. For all of the cases, characteristic Raman modes of PANI molecules such as C–H deformation vibration of a quinonoid ring at 1169 cm^− 1^, C-N stretching in polaronic structures at 1410 cm^− 1^ and C-C stretching vibrations of the semi-quinoid ring at 1595 cm^− 1^ are easily distinguishable^69–71^. The zigzag case (Fig. 6b, left panel) demonstrates isotropic behavior as the polarization angle varies from − 90° to 90° with a step size of 10°. In contrast, when linear plasmonic particles are illuminated along the chains at a 0° angle, super-radiant modes^52^ are excited, which provides a higher SERS signal of PANI shell compared to perpendicular polarization (Fig. 6b, central panel). For such 1D linear chains, the polarization sweep is carried out in discreet steps of 15°. However, this anisotropic behavior is not observed for random cases (Fig. 6b, right panel), where the random distribution of plasmonic particles results in fewer fluctuations in SERS signals for rotation of polarization angle (also at discreet steps of 15°). Furthermore, FDTD simulations revealed that the arrangement of plasmonic particles in opposite directions with mirror symmetry in the zigzag patterned case generates nearly identical extinction spectra. Consequently, this type of structure yields an isotropic SERS response. Although the degree of anisotropy in SERS response changes for linear particle chains at different excitation wavelengths, both zigzag and random cases maintain isotropic behavior for 532 and 785 nm excitations. (Figure S9) To accurately compare the SERS performances of these structures, single Raman measurements are taken from 10 different points, and the average SERS signal is calculated. The zigzag patterned case demonstrates the highest SERS signal at 633 nm excitation (Figure S10a), consistent with the calculated electric field profiles of such structures. Furthermore, the ordered structures show superior relative standard deviation (RSD) values compared to randomly arranged plasmonic particles (Figure S10b), and the SERS mapping of the 2D zigzag structure revealed homogeneous Raman signal distribution (Figure S10c). Thus, the zigzag patterning of plasmonic particles effectively combines the advantages of having random and linear structures. As a result, the direction of nanoparticle assembly chains no longer needs to be adjusted to optimize SERS performance, as the Raman intensity remains consistent regardless of sample orientation.

### Modulation of resonant modes through PANI exchange

**Fig. 7:**
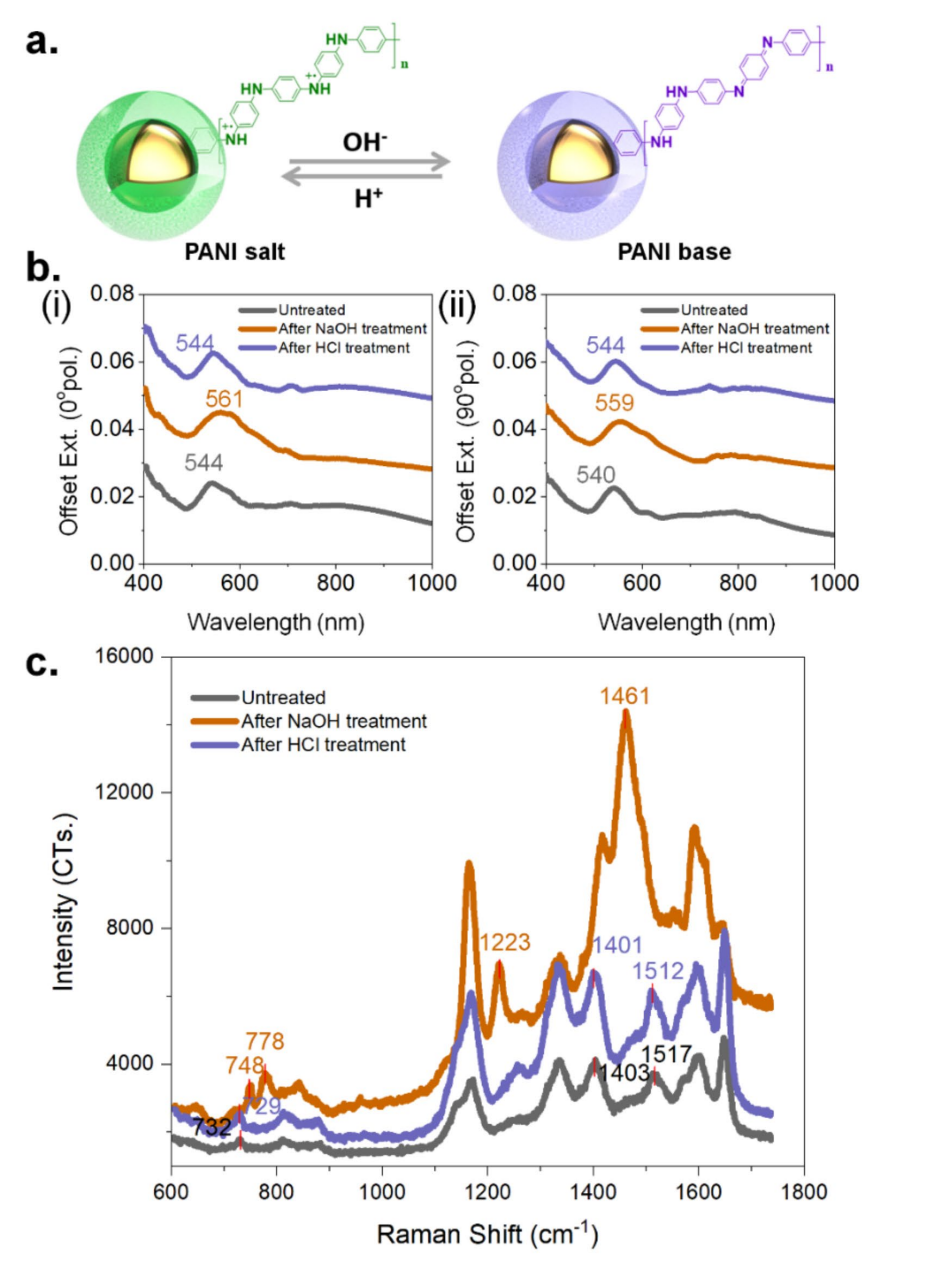
Conversion of the PANI coating between salt and base state. (**a**) The addition of NaOH shifts the PANI to a base state, altering the coupling conditions. However, this change is reversible upon the addition of hydrochloric acid (HCl). (**b**, i-ii) The transition between PANI states is monitored via extinction measurements across both polarization configurations. (**c**) SERS measurement taken after sequential NaOH and HCl treatments.

An important advantage of the PANI-coated AuNP used throughout our assembly processes lies in tuning the plasmonic resonance, where the redox-responsive characteristics of PANI modulate the refractive index, thereby enabling control over the resonant wavelength for these gold nanoparticles. By immersing the AuNP assembly in an alkaline or acidic aqueous solution for half an hour, the base state and salt state of PANI can be achieved and cycled (Fig. 7a). From PANI salt state to base state, the plasmonic peak of the zigzag assembly experienced an obvious red shift (from grey curves to orange curves in Fig. 7b). Subsequently, with alkaline treatment for half an hour, the plasmonic peaks moved back (blue curves in Fig. 7b). Under illumination with different polarization directions, the extinction spectra of the zigzag assembly are largely consistent.

SERS measurements of the zigzag assembly under acidic and alkaline conditions also revealed its plasmonic tunability and reversibility corresponding to PANI oxidation and doping states. In an acidic environment, PANI is in a protonated, doped state, where the nitrogen atoms in the polymer chain carry a positive charge. In alkaline environments, PANI loses its protonation and transitions to an undoped state. From Fig. 7c, we can observe that after treatment with NaOH, the SERS spectra undergo significant changes in both peak positions and intensities. Notably, the peak at 1461 cm⁻¹ corresponds to the vibration of the C = N or C = C bond with high conjugation^72^. After subsequent treatment with HCl, this peak moved to 1512 cm⁻¹ with an obvious decrease in the SERS intensity, indicating the reduced conjugation and PANI switched to its doped state.

## Conclusion

In this study, we have successfully demonstrated the fabrication of 2D zigzag wrinkle patterns and their application as templates for producing plasmonic zigzag chains as an efficient platform for isotropic SERS enhancements in comparison to pre-established 1D chains and randomly distributed particles. AFM characterization revealed detailed structural features, showcasing varied periodicities and gap widths. The influence of several experimental parameters on the morphology of the prepared 2D wrinkles was extensively analyzed, with particular emphasis on plasma treatment time, which significantly affects wrinkle wavelength and homogeneity. Additionally, the stretching ratio in two directions was found to play a crucial role in controlling wrinkle spacing, especially under asymmetric stretching in two directions, where the release sequence influences the orientation of the 2D wrinkles. A limit to the stretch ratio was observed; excessive variations lead to persistent cracking in the wrinkles. Based on these findings, the optimal conditions for creating templates for nanoparticle assembly were identified as 20%-15% stretching ratios in two directions with a 60-second plasma treatment. Polymer-coated Au nanoparticles were successfully assembled into the wrinkles using the confinement assembly process, where the zigzag and linear chains were observed through scanning electron microscopy. Optical characterization, supported by FDTD simulations and UV-Vis-NIR spectroscopy, indicated that zigzag arrangements offer comparable and, in some cases, superior plasmonic responses compared to traditional linear arrays. This is particularly evident in their application in SERS, where the zigzag arrays show distinct advantages in isotropy and signal enhancement through the incorporation of polarization-insensitive plasmonic hotspots. Thus, the insights gained from the isotropic and anisotropic configurations of nanoparticle arrays have important implications for designing next-generation materials by combining the benefits of nano-scale precision with macro-scale functionality. Moreover, the adaptability of the PANI-coated AuNPs demonstrated through their tunable plasmonic response to environmental refractive index changes highlights the potential of smart nanoparticle systems with dynamic control, opening avenues for innovations in smart materials and responsive technologies.

## Electronic supplementary material

Below is the link to the electronic supplementary material.

Supplementary Material 1

## Data Availability

Data generated or analyzed during this study are available upon reasonable request. For more details, please contact the corresponding author Ziwei Zhou.
