# Supplementary material for: 2D wrinkle assisted zigzag plasmonic chains for isotropic SERS enhancement
Source: Sci Rep. 2025 Jan 29;15:3662. doi: 10.1038/s41598-025-87504-8 (PMC11779806; doi:10.1038/s41598-025-87504-8)
Supplement: Supplementary file 1 — Supplementary Material 1 [file 41598_2025_87504_MOESM1_ESM.docx]

Supplementary Information

**2D Wrinkle Assisted Zigzag Plasmonic Chains for Isotropic SERS Enhancement**

*Ziwen Yu,*1,2,‡ *Swagato Sarkar,*1,‡ *Sezer Seçkin,*1,‡ *Ningwei Sun,*3 *Anik Kumar Ghosh,*4 *Sven Wießner,*2,4 *Ziwei Zhou*1,∗ *and Andreas Fery,*1,5,6,∗

1Leibniz-Institut für Polymerforschung Dresden e.V. (IPF), Institute of Physical Chemistry and Polymer Physics, 01069 Dresden, Germany

2Institute of Materials Science, Technische Universität Dresden, 01062 Dresden, Germany

3Leibniz-Institut für Polymerforschung Dresden e.V. (IPF), Institute of Macromolecular Chemistry, 01069 Dresden, Germany

4Leibniz-Institut für Polymerforschung Dresden e.V. (IPF), Institute of Polymer Materials, 01069 Dresden, Germany

5Center for Advancing Electronics Dresden (cfaed), Technische Universität Dresden, 01069 Dresden, Germany

6Chair for Physical Chemistry of Polymeric Materials, Technische Universität Dresden, 01062 Dresden, Germany

‡These authors contributed equally

∗Corresponding authors: [zhou@ipfdd.de,](mailto:zhou@ipfdd.de) [fery@ipfdd.de](mailto:fery@ipfdd.de)


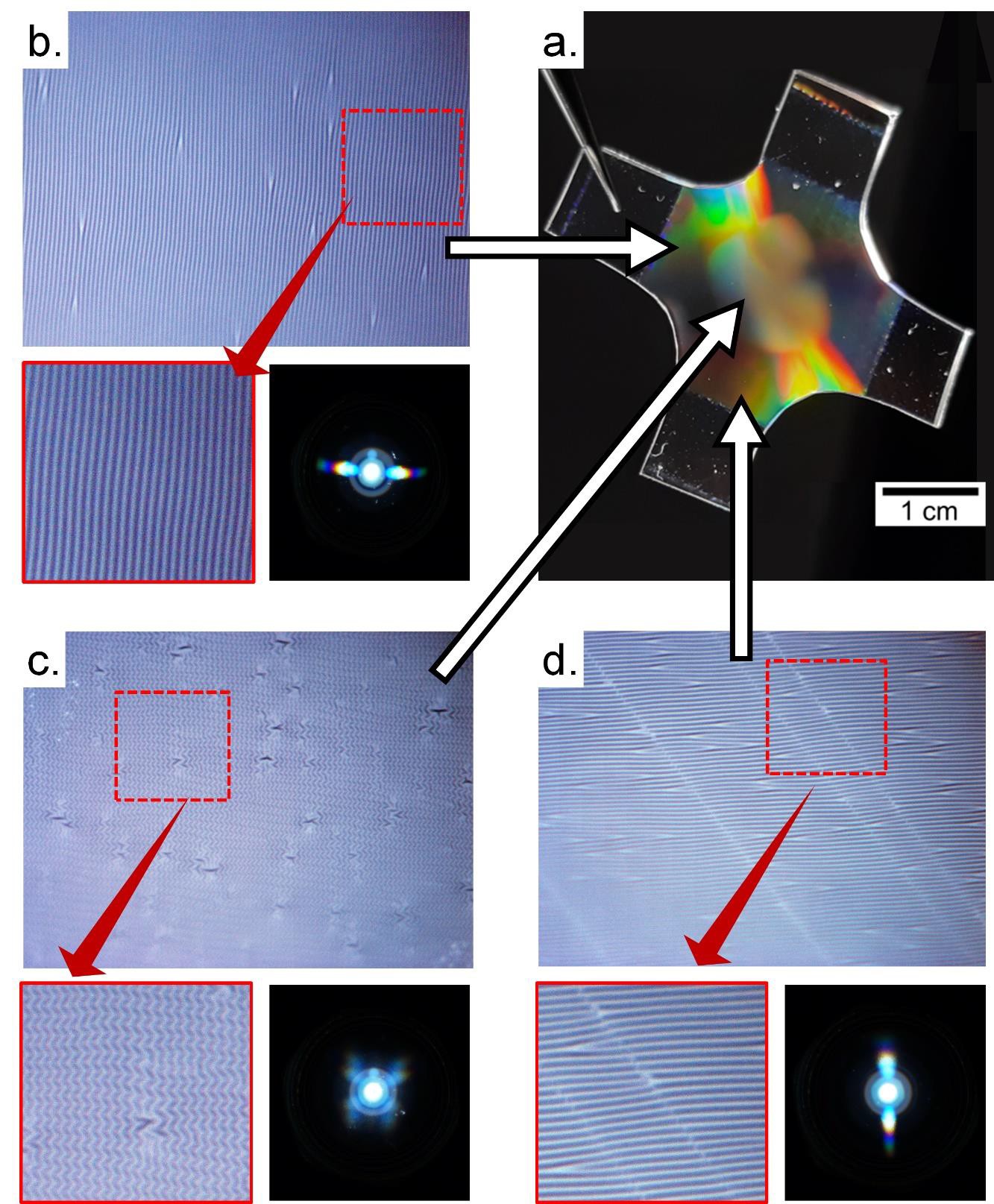


**Fig. S1.** Microscopic investigations at different positions of a 2D PDMS wrinkle.

**a.** Photograph of a 2D wrinkled PDMS substrate after strain release. Investigations are conducted at three positions, showing **b, d.** 1D linear wrinkles at the two arms of the cross- shaped substrate and **c.** 2D zigzag wrinkles at the center. For each of these positions, large- scale bright-field images, magnified views showing wrinkling orientation, and diffraction orders using a Bertrand lens setup are presented.


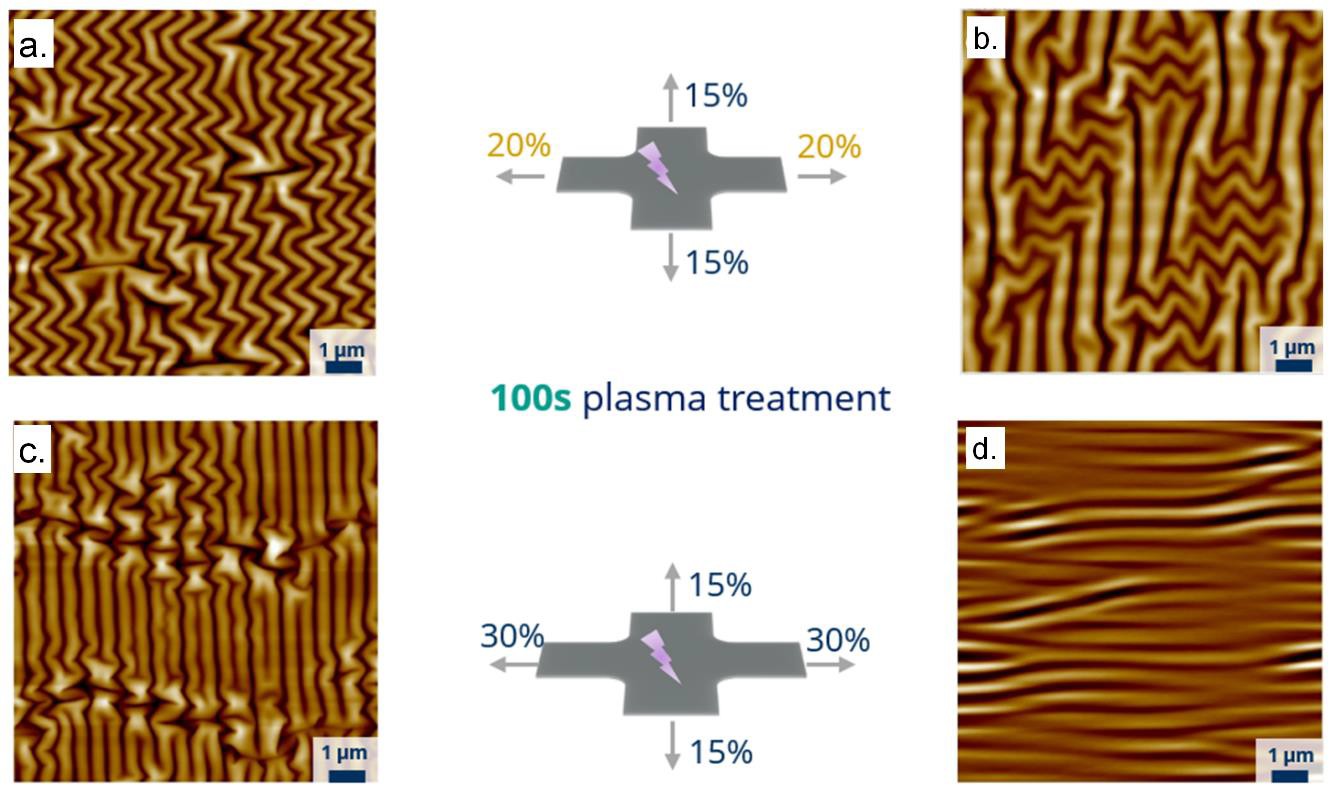


**Fig. S2.** AFM images of 10 µm areas and the corresponding schematic diagrams describing the direction of wrinkles with asymmetric stretching ratios under 100 s plasma treatment.

a. 20% − 15% stretching ratios — relaxing of the larger strain first. b. 20% − 15% stretching ratios — relaxing of the smaller strain first. **c.** 30% − 15% stretching ratios — relaxing of the larger strain first. **d.** 30% − 15% stretching ratios — relaxing of the smaller strain first.


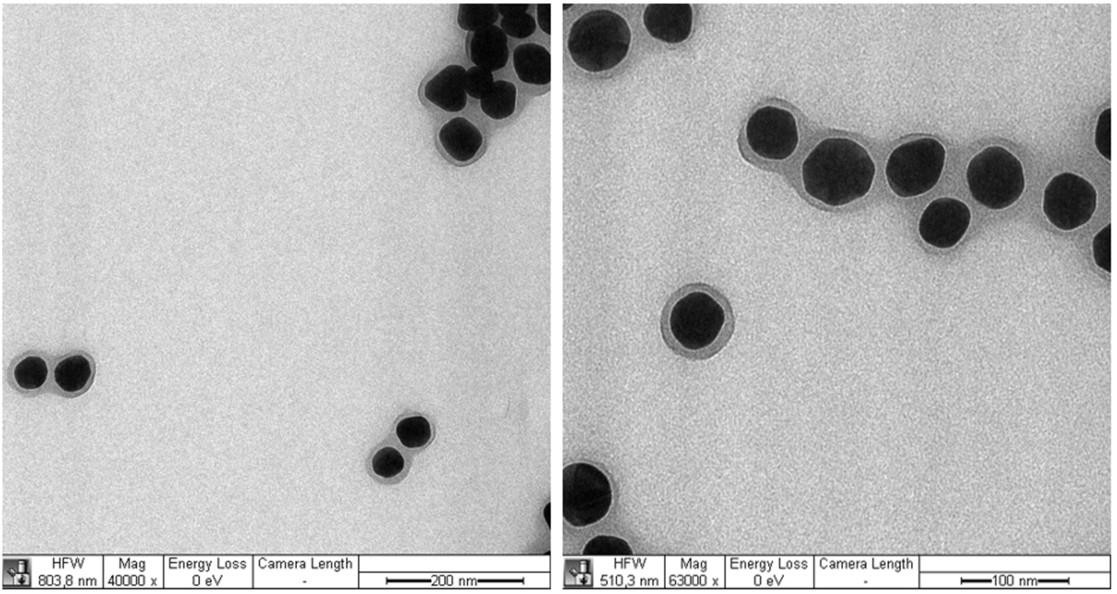


Figure S3. TEM images of the as-prepared PANI-coated AuNPs at different magnifications.


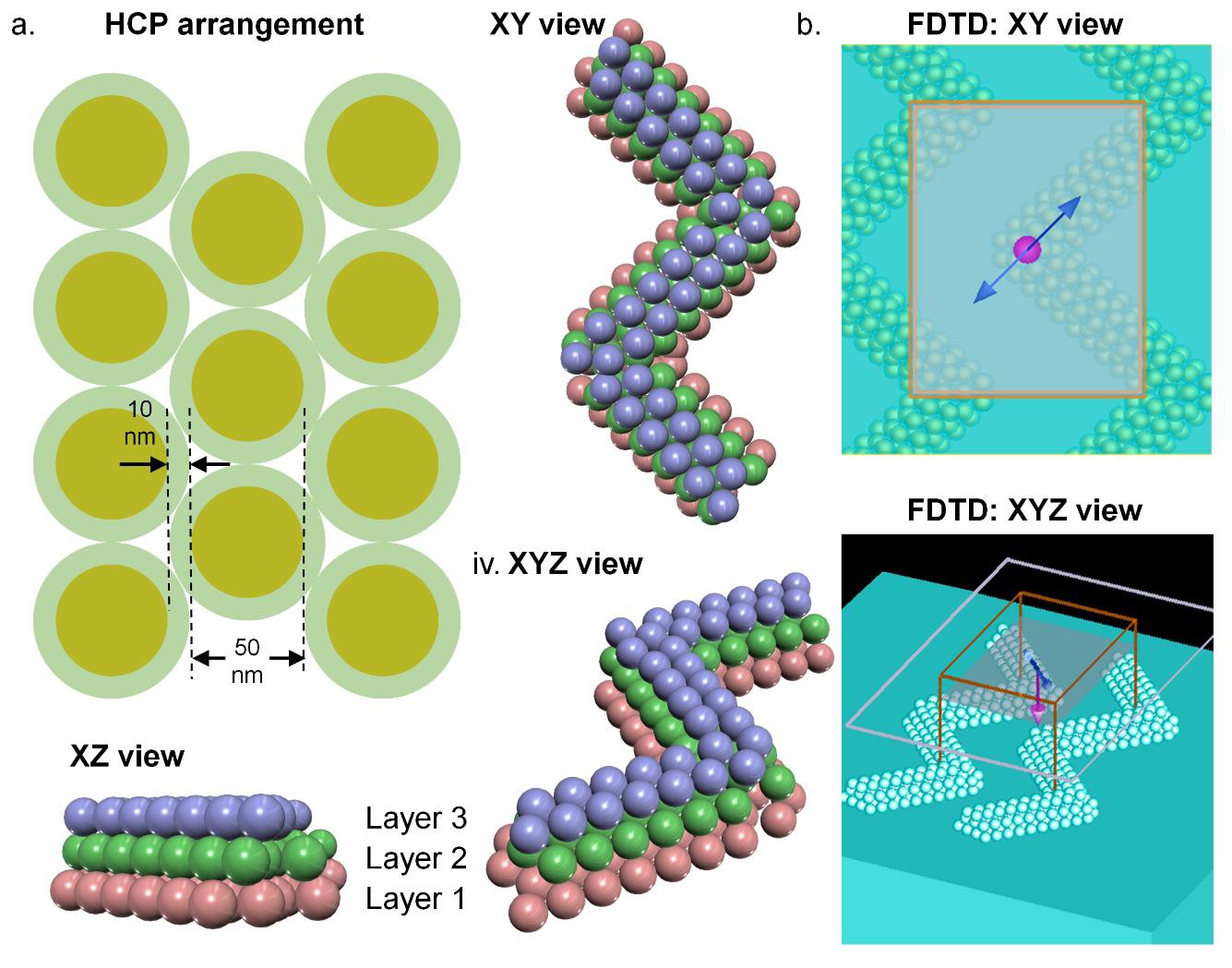


**Fig. S4.** Hexagonal close-packed (HCP) arrangement of 70 nm diameter AuNP@PANI. **a.** XZ view, XY view, and XYZ view of the 3D stacked zigzag chains. **b.** The corresponding XY and XYZ views of the FDTD unit cell in the simulation software.


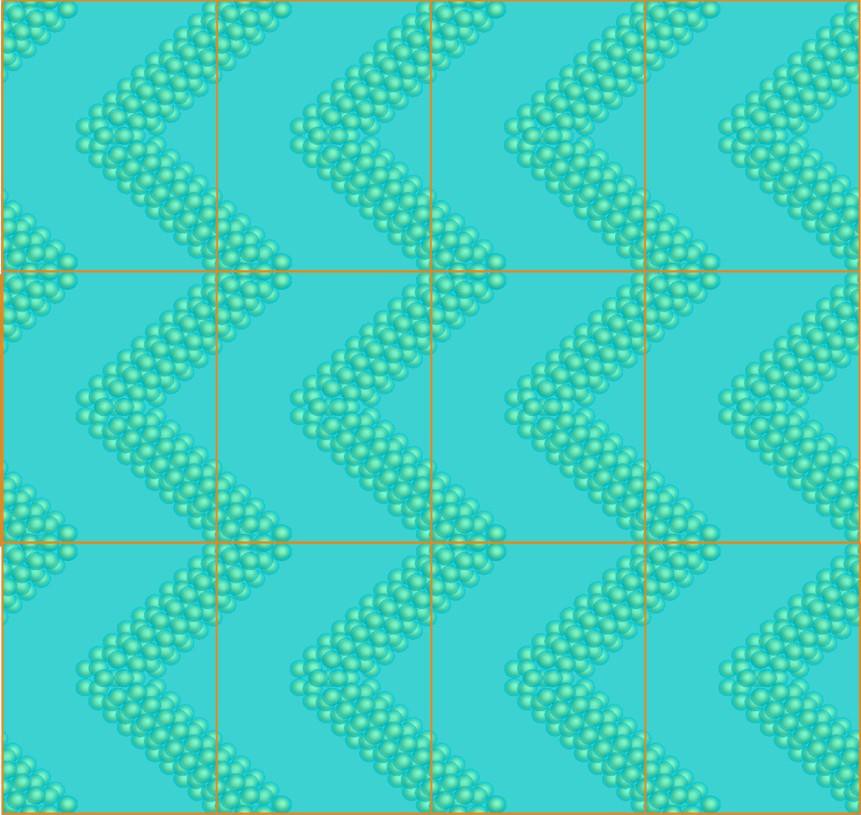


**Fig. S5.** The FDTD unit cell, with an X span of 750 nm and a Y span of 1040 nm, replicates the desired 2D periodic zigzag array with features resembling experimentally observed configurations when repeated in the X and Y directions.


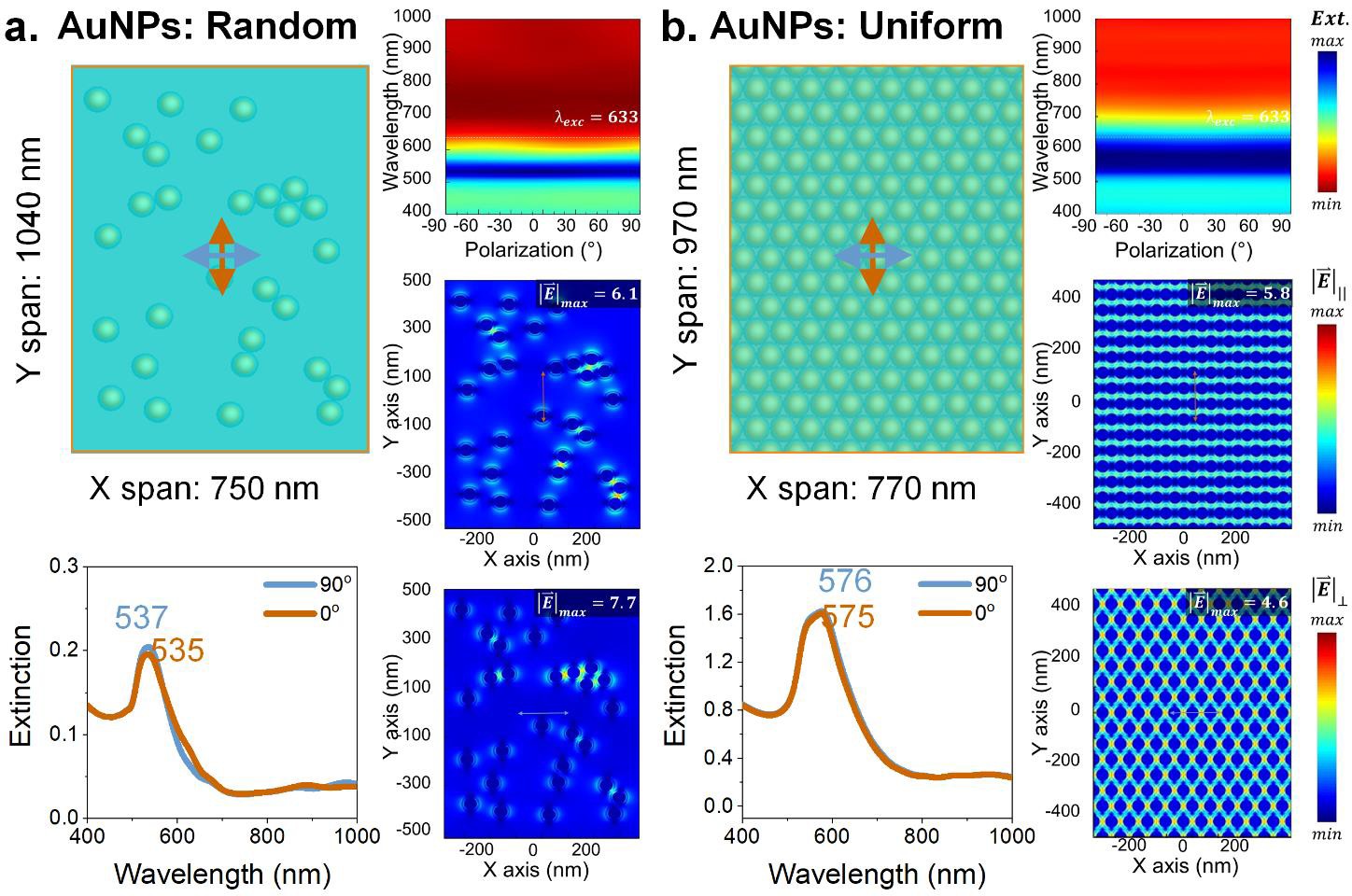


**Fig. S6.** FDTD comparison of (a) randomly and (b) uniformly distributed AuNPs, in terms of their unit cell, optical extinction at 0◦ and 90◦ angles, optical extinction as a function of polarization angle sweep, and electric field distribution at two orthogonal polarization states.


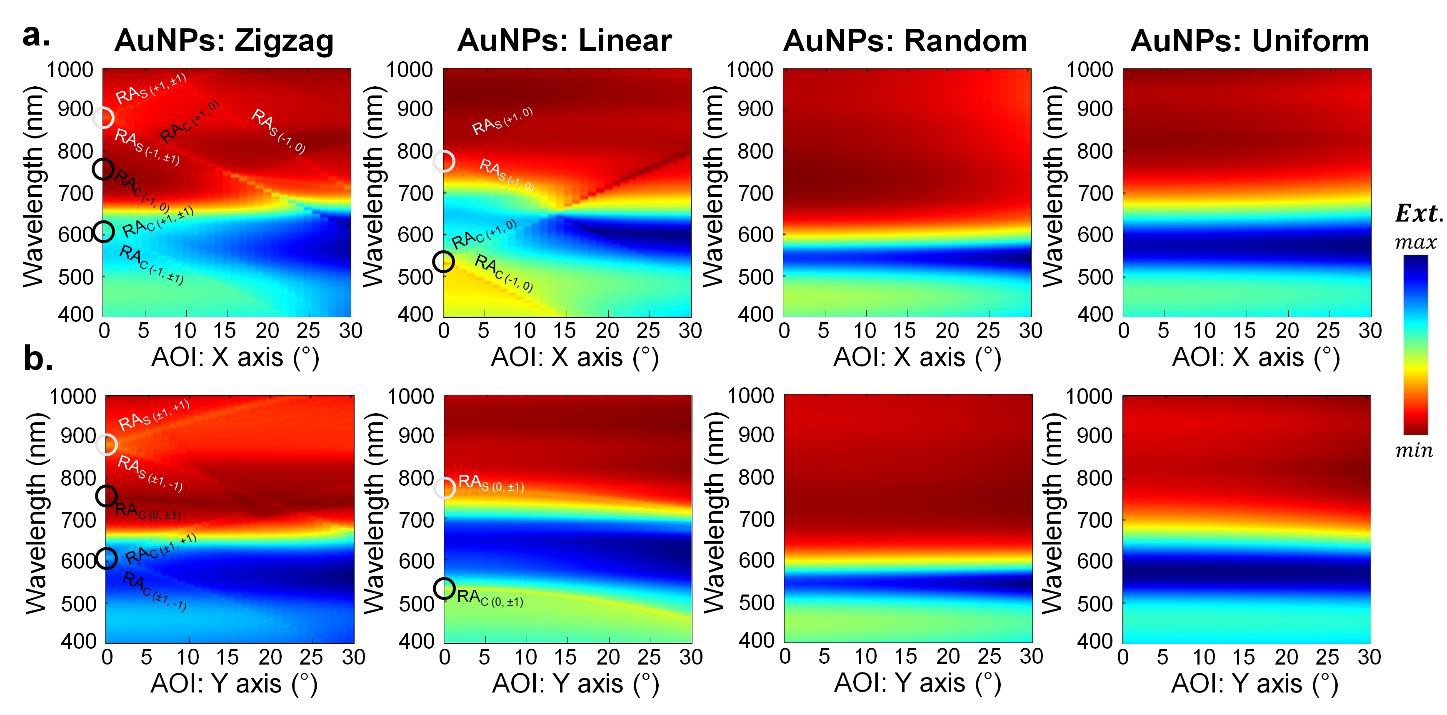


**Fig. S7.** FDTD-based comparison of extinction spectra for variation of angle of incidence (AOI) along the **a.** X and **b.** Y axes for various configurations of 2D periodic zigzag chains, 1D periodic linear chains, randomly distributed AuNPs, and a uniform monolayer of AuNPs. For the 2D and 1D periodic configurations, the diffraction-associated Rayleigh Anomalies (RAs) corresponding to the substrate (S) and cover (C) are identified.


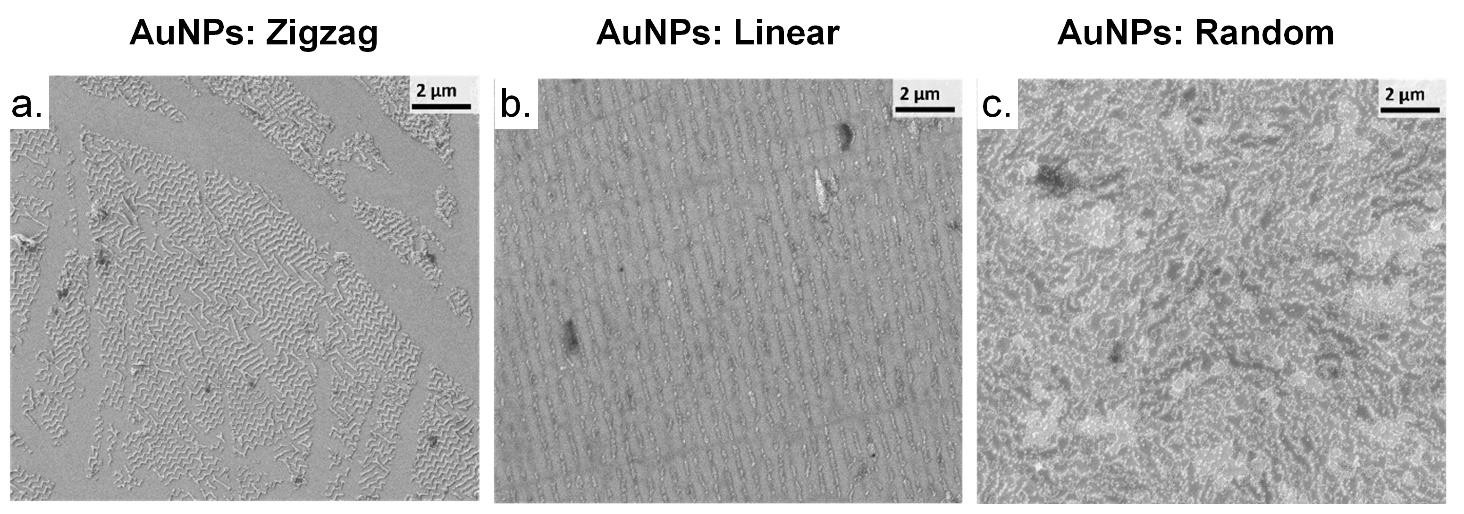


**Fig. S8.** SEM image showing a large-area assembly of PANI-coated AuNPs. **a.** Assembly of zigzag chains; **b.** Assembly of linear chains; **c.** Assembly of random chains. These images illustrate the capability to accommodate varying particle sizes in each assembly.


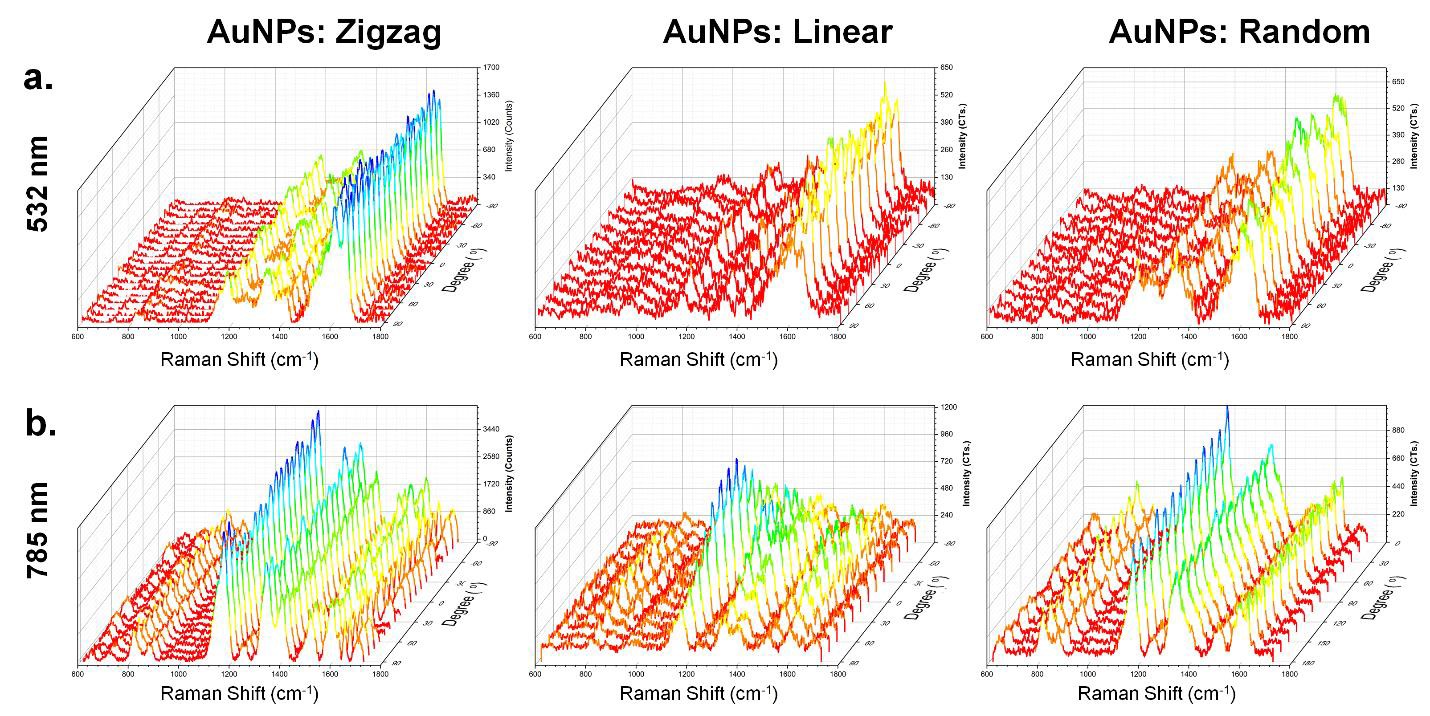


**Fig. S9.** Comparative analysis for SERS enhancement with various laser excitation wavelengths. (a, i-iii) The corresponding Raman shifts from three arrangements of PANI- coated AuNPs under 532 nm laser excitation. (b, i-iii) The corresponding Raman shifts from three arrangements of PANI-coated AuNPs under 785 nm laser excitation.


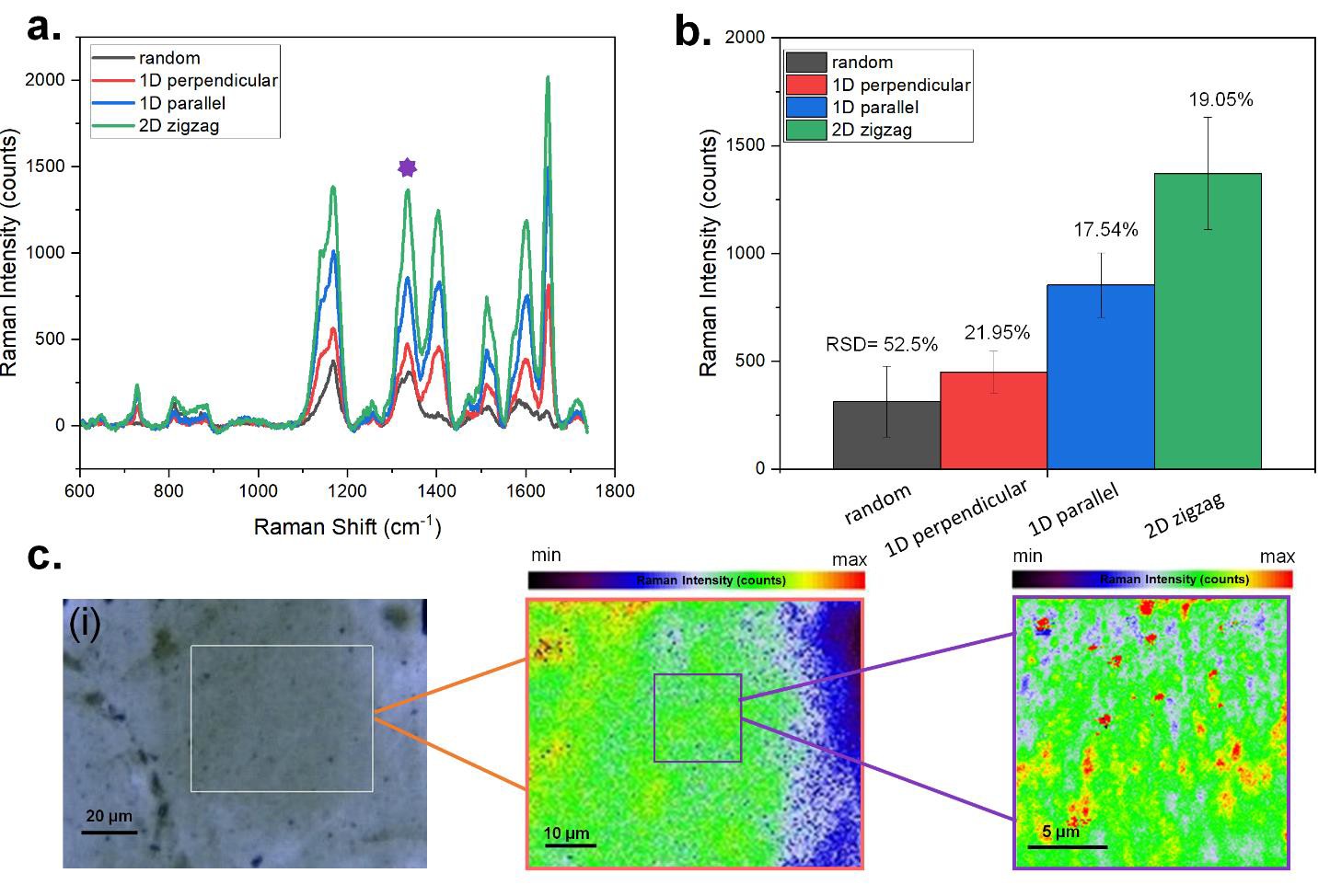


Figure S10. Comparative analysis of SERS enhancement with 633 nm laser excitation. a) The spectra for random distributions, 1D lines, and 2D zigzag chains of AuNPs are calculated by averaging single measurements from 10 different positions. Among these configurations, the 2D zigzag chain exhibits the maximum enhancement for the PANI SERS signal. The Raman mode at 1336 cm−1 is highlighted for further discussion. b) The mean intensities of the different structures at 1336 cm−1 are presented with error bars representing the standard deviation. To facilitate comparison, the relative standard deviation (RSD), calculated as the ratio of the standard deviation to the mean value, is displayed on top of each bar. c) SERS mapping of the 2D zigzag sample. Left: The optical image of the 2D zigzag sample that was SERS mapped using a 633 nm excitation wavelength. Center: Mapping performed with a 20X magnification objective. Right: Mapping performed with a 100X magnification objective.


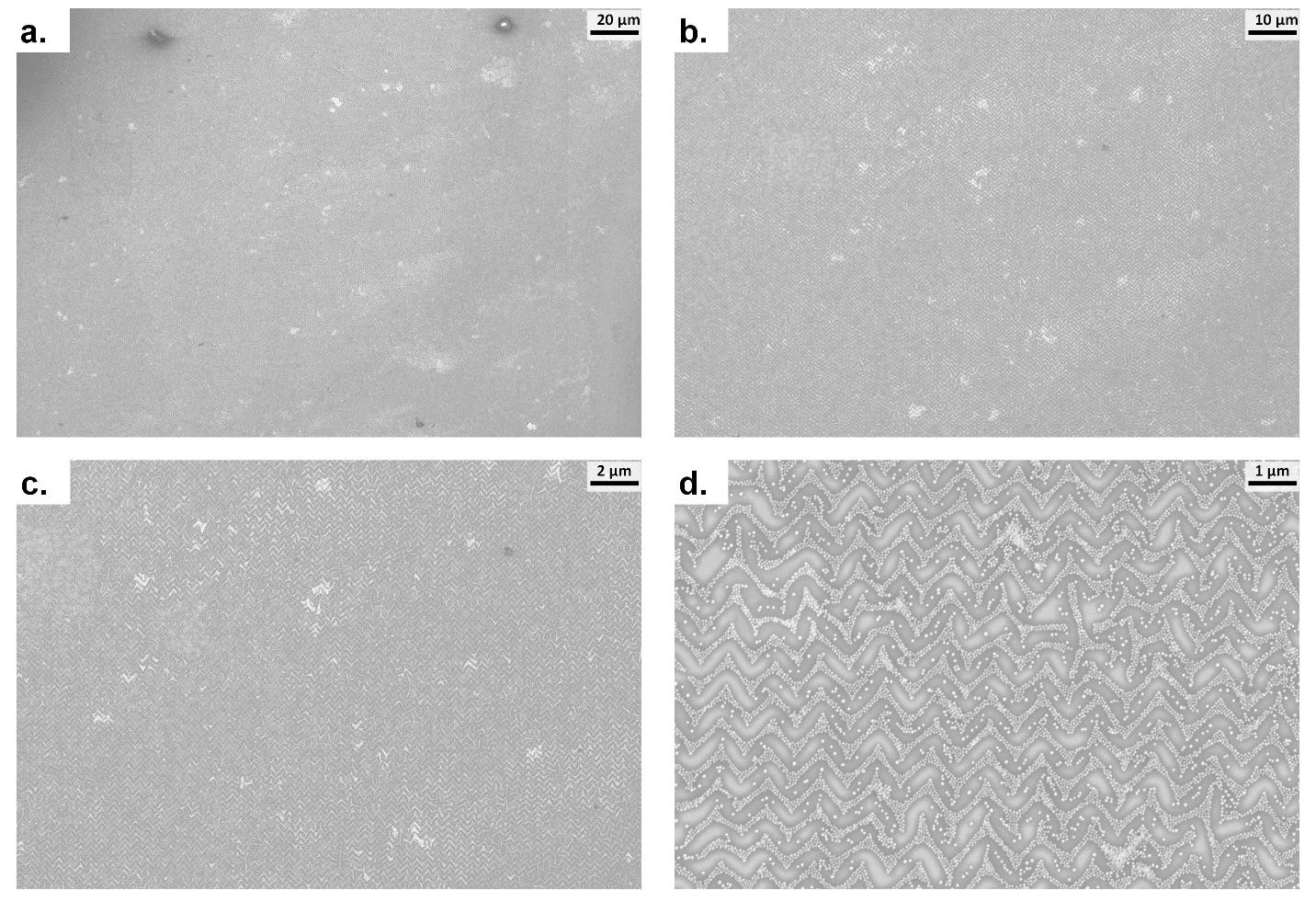


Figure S11. SEM images of a 2D zig-zag assembly of PANI-coated AuNPs, demonstrating uniform coverage over a large area. Panels (a-d) show the structure at four different magnifications.
